# Plasmonic Probe With Circular Nano-Moat for far-Field Free Nanofocusing

**DOI:** 10.1186/s11671-016-1619-y

**Published:** 2016-09-22

**Authors:** Mingqian Zhang, Tianying Wang

**Affiliations:** 1 Qian Xuesen Laboratory of Space Technology, China Academy of Space Technology, Beijing, 100094 China; 2 The Hong Kong Polytechnic University, Hung Hom, Kowloon Hong Kong

**Keywords:** Surface plasmon polariton, Scanning near-field optical microscopy, Plasmonic probe, Nanofocusing

## Abstract

In this work, a metallic probe with a sharp tip and two half-circular nanostructures on its base is introduced and investigated. The proposed design aims at improving the detection performance of a probe for scattering scanning near-field optical microscopy in terms of enhanced signal-to-noise ratio. Under the premise of processing feasibility, the structure of the probe is designed and optimized with three-dimensional finite-difference time-domain method. And then the performance and optical property of the probe are theoretically investigated and experimentally demonstrated using a scanning near-field optical microscope with aperture probe. It is indicated that a tightly confined optical field with significantly reduced far-field background can be achieved at the tip apex of the probe.

## Background

Scanning near-field optical microscopy (SNOM) is a promising method for analyzing and measuring the optical property of matter at the nanoscale. It provides a wealth of optical information with nanoscale spatial resolution. With its rapid development in recent decades, it has been applied to a wide range of scientific research including DNA imaging in biomedical science [1], nanoparticles probing [2], and nanotubes detection [3, 4] in chemical science and the light-inducement of nano-movement [5] in physical science.

A scattering SNOM (s-SNOM) is a form of SNOM. Its advantages over the aperture SNOM are the higher resolution limited essentially only by the size of the probe apex [6] and the potential to enhance the local optical signal with tip-enhancement. A typical s-SNOM setup includes incident light, a scattering probe, excitation/collection optical path, a detector, sample-probe distance regulation system, and scanning mechanism. The optical field in a nearby region of an illuminated sample consists of both propagating and evanescent field components. In an s-SNOM, the evanescent field which contains the detailed optical information of the sample can be converted into propagating radiation by use of a scattering probe [4, 5]. Ultimately, the optical image is formed with high-resolution optical information in the radiation observed by the detector. The metallic tip with a typical size of several nanometers at its apex is arguably the critical component of an s-SNOM setup. It acts as an optical antenna that efficiently converts propagating optical field into localized energy and vice versa [5]. Lately, several approaches have been proposed to improve s-SNOM performance by the optimization of the tip. Among them, certain research interest has been devoted to the improvement of the tip preparation process [7–9]. And some other researchers have been focused on the optimization of tip design [10–12]. Since the near-field optical signal can be inherently weak, it is favored that the probe’s is capable of generating far-field free nanofocusing and collecting high signal-to-noise ration evanescent field.

The motivation of this paper is to develop and investigate the characterization of a micro-fabricated optical antenna that strengthens the local electromagnetic field and the absolute scattering efficiency leading to enhance the imaging resolution of s-SNOM. Through the experiments, optical antennas with different geometrical shapes were examined and compared.

## Methods

**Fig. 1:**
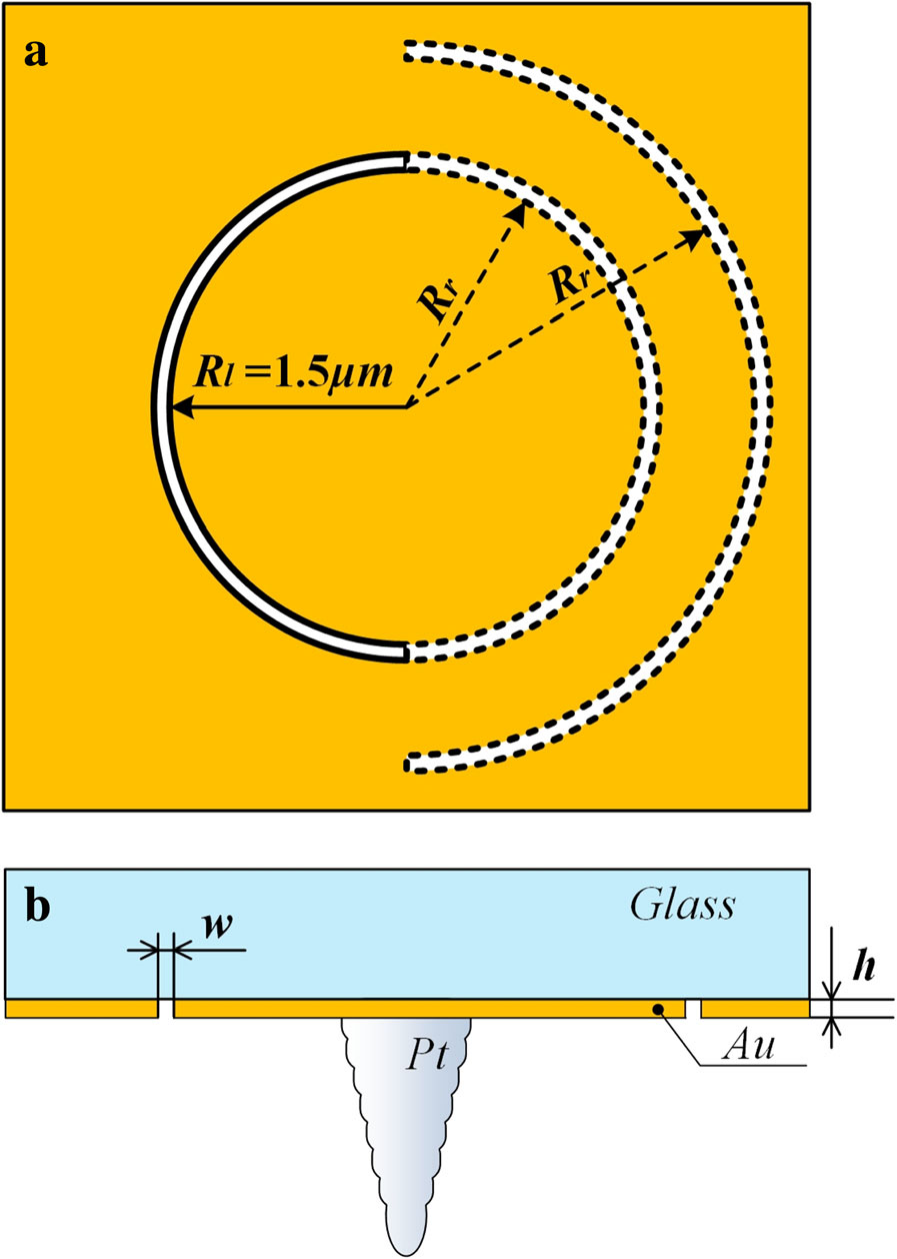
Schematic top view (**a**) and front view (**b**) of the designed s-SNOM probe

In this research, we focus on improving the detection sensitivity of an s-SNOM probe by isolating the incident light from the local detection area to reduce the far-field background. Based on this idea, a metallic probe, as shown in Fig. 1, with a sharp tip and nanostructures on its base has been designed. In this design, a circular nano-moat, or a symmetry breaking nano-circular slot was utilized to achieve phase delay and matching. This kind of nanostructure was firstly proposed for in-plane surface plasmon polariton (SPP) focusing by Fang et al. [13, 14]. Here, it was used to launch SPP at metal-dielectric interface and composite the phase difference for focusing at the sharp tip, as the tip guides SPP propagating through the surface.

The finite-difference time-domain (FDTD) method was introduced to numerically investigate the surface plasmon-related optical property and performance and to theoretically optimize its structure. A p-polarized plane wave with the wavelength of 632.8 nm was used as the excitation light. It was incident from the glass side on the base of the probe at normal incidence. The probe was composed of a sharp metallic tip and a gold-coated base with a couple of semi-circular slots corrugated on it. Two homocentric semi-circular slots with the same width of 0.10 μm were carved through the 0.12-μm-thick gold film. The thickness of the gold film was much thicker than the skin depth. Hence, the direct incident light was screened out to avoid the immediate exposure of the specimen to the incident light, so that the far-field background can be significantly reduced consequentially. The metallic slots in circular shape performed as a plasmonic launcher to couple the incident light into the propagating SPP waves and a sink to guide the SPP waves to converge toward the center. The radius of the left semi-circular slot was fixed to 1.50 μm and the right one was numerically optimized with parameter sweep. The radius mismatch was introduced to composite the phase difference. A platinum tip with the height of 1.50 μm stood at the center of the semi-circular slots on the gold film. To account for the realistic processing capacity, the tip was composed of a set of platinic pies of decreasing size stacked one on top of another resembling a cone. The diameter at the bottom of the tip was 0.80 and 0.25 μm at the apex.

To verify our simulation results, the optical near-field detection was performed. The probes were experimentally prepared as follows. First, a 0.12-μm-thick gold film was coated on a glass substrate. Secondly, a couple of sub-wavelength slots with the same width of 0.10 μm were carved through the gold layer by focused ion beam (FIB) milling. Ultimately, at the center of the semi-circular slots, a cone-like platinum was locally deposited via gas-assisted FIB deposition.

For the detection of the optical field, an upright SNOM (NTEGRA Spectra, NT-MDT, Russia) in collection mode was used to directly measure the sub-wavelength focusing of the local electromagnetic field at the tip apex. Cantilever probes with nano-apertures were employed to collect the optical near-field signal. The high-resolution intensity distribution at the probe apex was mapped in a plane parallel to the probe base.

## Results and Discussion

The simulations using FDTD method were conducted. The radius of the right slot was swept from 1.50 to 2.20 μm with the intervals of 0.10 μm to optimize the probe structure. The electric field intensity distributions at the position 5 nm below the tip apex were measured.

**Fig. 2:**
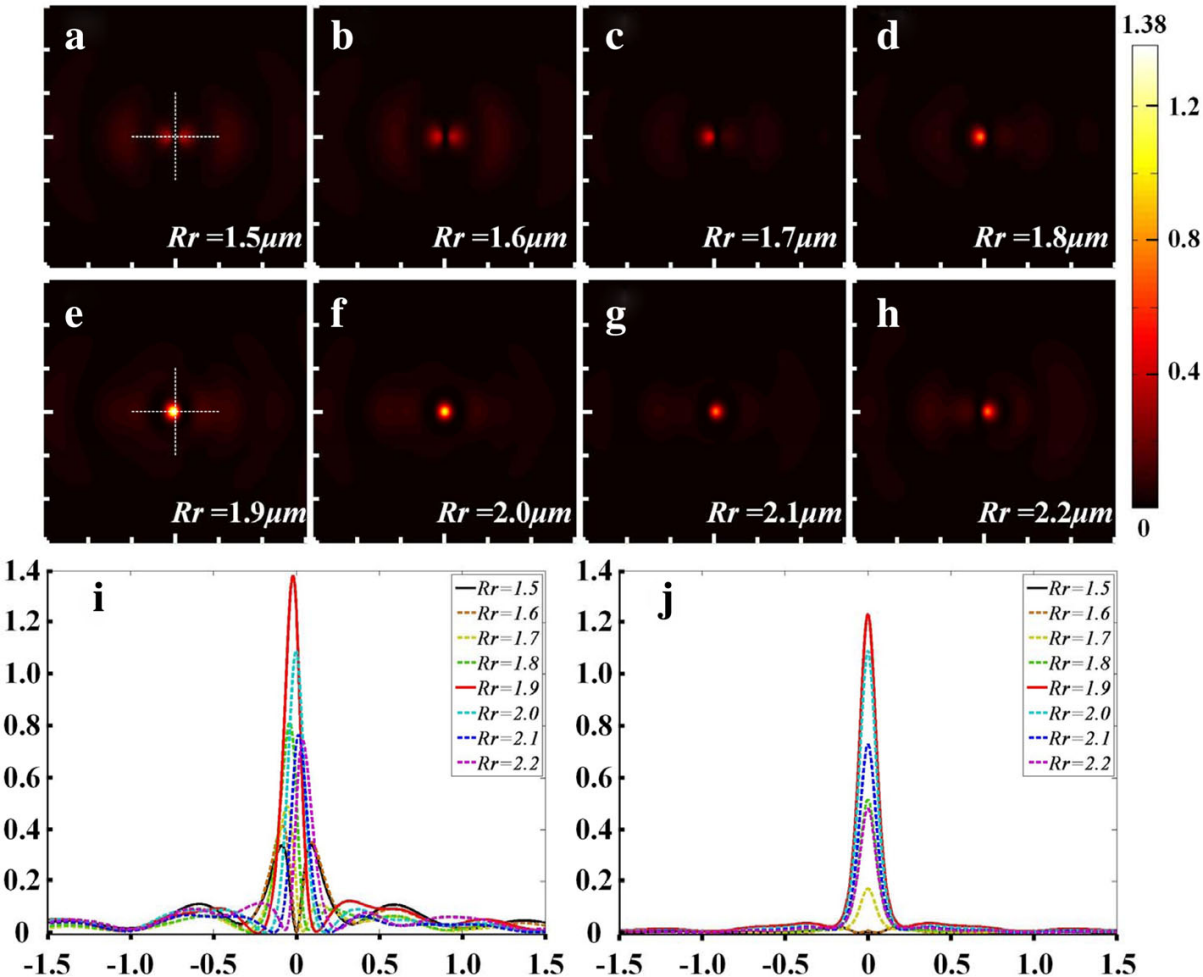
Field intensity distribution variation with right slot radius. **a**–**h** Horizontal section at 5 nm below the probe apex, right slot radius caries from 1.50 to 2.2 μm. Normalized x- (**i**) and y-cuts (**j**) of the field intensity right below the probe apex

The simulation results are presented in Fig. 2. Figure 2a–h demonstrates the intensity distributions with the variation of the radius of the right slot. Besides the relatively weak side lobes, the major distribution of the optical field gradually varies from two roughly symmetric side lobes to one solid facula located right below the tip. Also, there is a decreasing tendency in the optical field intensity around the tip apex and the strongest center moves from left to right when the radius increases from 1.90 to 2.20 μm. Figure 2i, j shows the line cuts of the field intensity along the horizontal and the vertical axis, respectively. The intensities of different probes are plotted as functions of the displacement. They demonstrate the trends and changes in the field intensity with the variation of the right slot radius clearly. It could be concluded that the intensity is maximized when the radius of the right slot is 1.90 μm. The peak position is located slightly to the left of the center. And the spatial resolution of this probe could be revealed by full width at half maximum (FWHM) of the *x*-*z* and *y*-*z* cross section. The focus is asymmetric with FWHM along the horizontal and the vertical axis of 110 and 120 nm, respectively.

Particularly, the probe structures with right slot radiuses of 1.50 and 1.90 μm were selected for further comparative study. The probes in experimental group were modeled and fabricated according to the optimized structure design: one kind of probe has its slot structure formed by two halves of the circles with radius of 1.50 and 1.90 μm, respectively; the other that serves as a control group has its right slot radius of 1.50 μm. In other words, the plasmonic-launching structure of the second kind of probe is simply a circular slot with radius of 1.50 μm that is a rotational symmetric structure. More detailed simulations and direct experimental validations were carried out then.

**Fig. 3:**
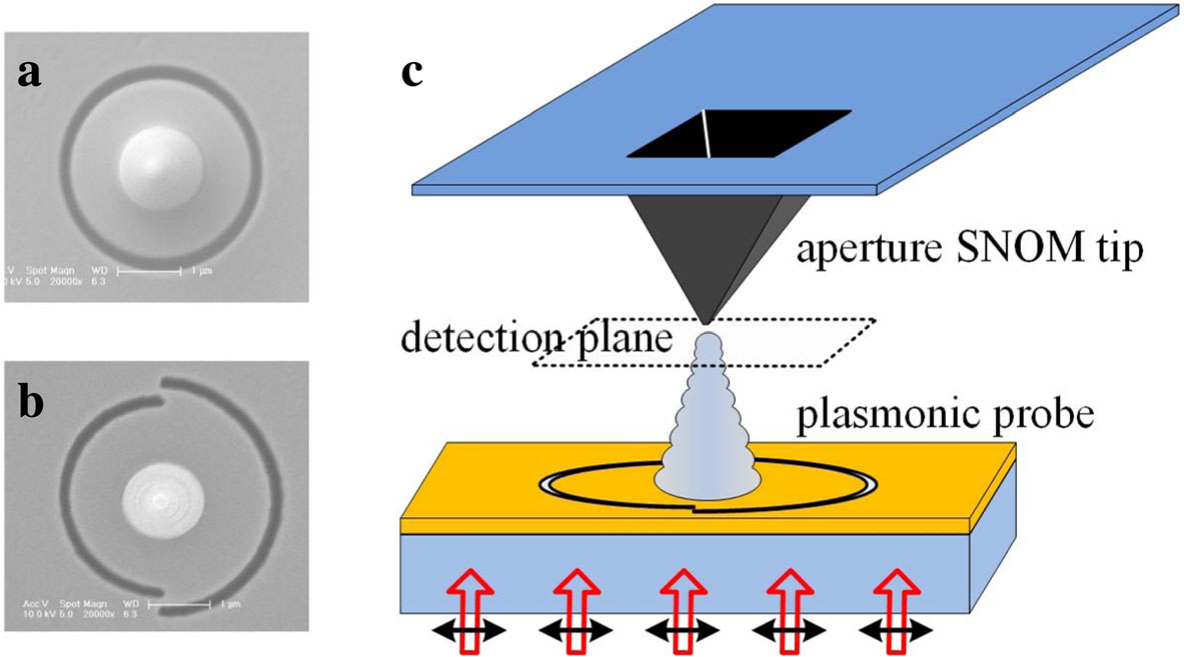
SEM images of the prepared probes (**a** and **b**) and diagram of the measurement scheme (**c**)

To verify the theoretical calculation, the two kinds of probes were fabricated. As shown in Fig. 3a, b, the only difference between them is the radius of the right slot. Then the local optical field distributions at the apexes of probes were directly measured, and the measurement scheme is shown in Fig. 3c. The inversely placed probe was illuminated from the base side by a linearly polarized red laser with the wavelength of 632.8 nm. The slot structure of the probe coupled the incident light into propagation SPP waves. And the SPP waves traveled along the air-metal interface on the base and the tip, and then encountered at the area around the tip apex. The intensity distribution at the probe apex was mapped in a plane located several nanometers away from the apex by a transmission-mode aperture SNOM. A 3 × 3 μm detection region centered at the tip apex had been selected and raster-scanned. Due to the direct incident light resistance feature of the sample probe, almost background-free intensity distributions of the electric field were experimentally obtained.

**Fig. 4:**
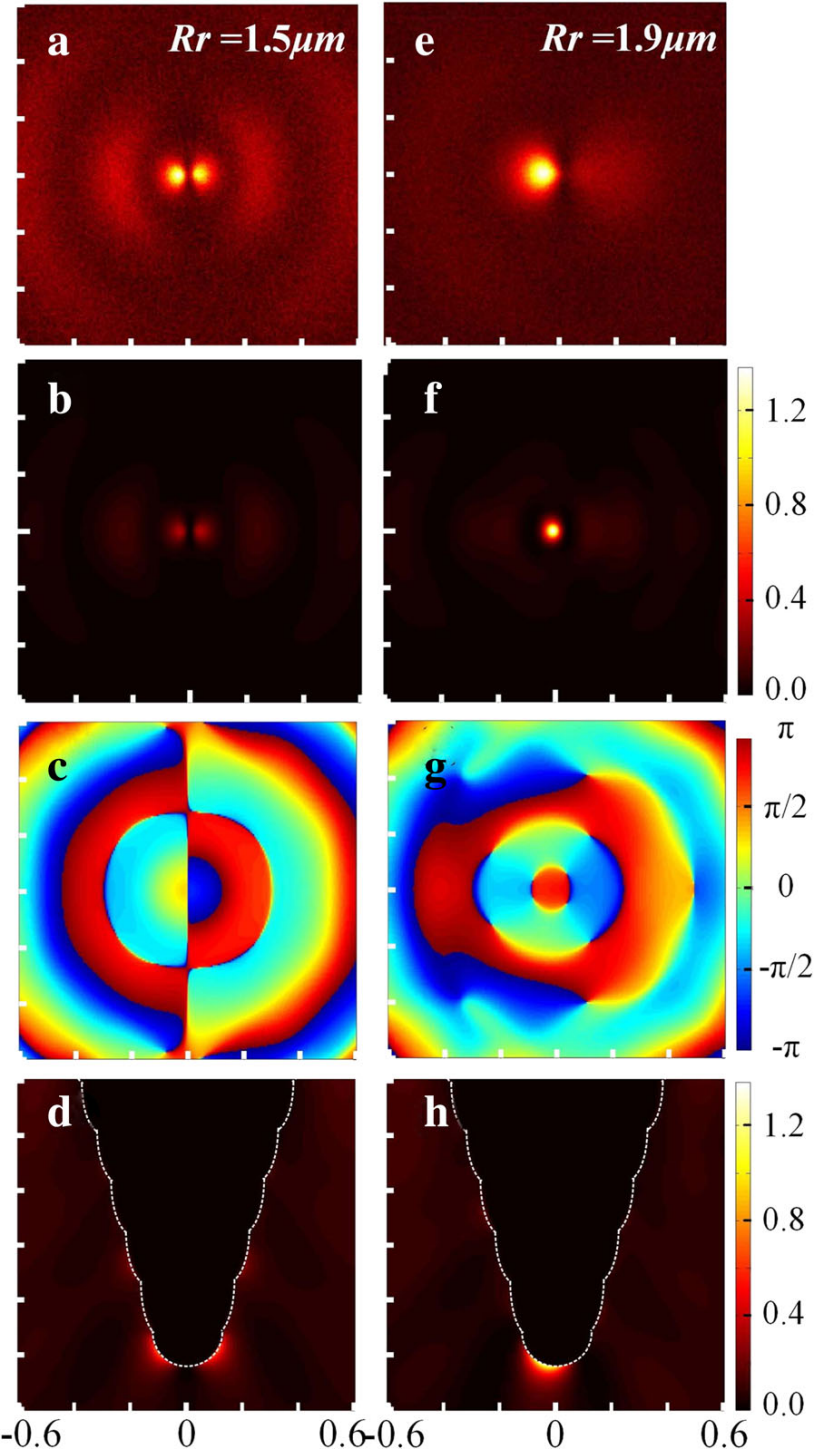
The left column (**a**–**d**) shows the experimental and simulation results of the probe with Rr = 1.50 μm, while the right column (**e**–**h**) illustrates that of Rr = 1.90 μm, (**a**) and (**e**) Measured and (**b**) and (**f**) calculated optical field distribution and (**c**) and (**g**) calculated phase distribution and (**d**) and (**h**) calculated cross section of the probe tip. The borders of the probe structure are depicted with *white dashed lines*

The experimental and simulation results of the two kinds of probes are presented correspondingly in Fig. 4 for intuitive comparison. The observed pattern of the probe in control group (Rl = Rr = 1.50 μm, shown in Fig. 4a) appeared to be two separate bright spots with a series of side lobes around. Such field distribution is not suitable for near-field excitation in a SNOM setup, as it may lead to imaging issues like ghosting. Compared with Fig. 4b, it could prove that the experimental result was in close agreement with the theoretical prediction. However, the measured intensity of left spot was relatively strong than the right one, compared to the simulation results, which might be attributed to the processing defects of the probe structure and the non-parallelism between the probe base and the detecting plane. Figure 4c demonstrates the calculated phase distribution of electric field in Z-direction. It could be observed that a split located roughly along the middle vertical axis divided the phase distribution into two opposite parts. It indicates that the phase of electric field in Z-direction turns to be mismatched, which is due to the non-rotational symmetry of the linearly polarized laser excitation and the symmetry of the probe structure. Consequently, the optical field distribution presented as two split light-spots distributed on the two sides of the tip apex (see Fig. 4d).

The right column shows the experimental and simulation results of the probe with Rl = 1.50 μm and Rr = 1.90 μm. It shows that an intense optical field was developed at the apex of the probe tip and the local field was highly confined to a tiny volume near the apex. On the whole, the experimental result agreed well with the simulation, although there was a slight broadening of the bright region in the measured optical field distribution.

The simulated phase image (see Fig. 4g) showed fairly a good electric field phase match at the center in Z-direction. And a beyond diffraction-limited nanofocusing was generated at the probe apex. Hence, this probe could be used as a SNOM probe to produce the nanometer scale illumination field with reduced far-field background.

## Conclusions

In summary, the simulations using 3D-FDTD and experiments have been conducted for measuring the optical properties of the s-SNOM probes with specific nanostructures on their bases. Despite negligible differences, it can be concluded that the results of the experiments agreed with the theoretical calculations. The electrical filed intensity distributions of probes with base nanostructures of various right slot radiuses have been analyzed and compared. Also, the optical properties of probes with either rotational symmetric or asymmetric circular nanostructure are compared, and the one with asymmetric nanostructure is found to more focused. Therefore, a probe with an asymmetric circular nanostructure (Rr = 1.90 μm and R_l_ = 1.50 μm) on its base engenders maximum electrical intensity at the tip apex and minimum far-field background when a polarized excitation light is used.

## Abbreviations

FDTD: Finite-difference time-domain
FIB: Focused ion beam
FWHM: Full width at half maximum
SNOM: Scanning near-field optical microscopy
SPP: Surface plasmon polariton
s-SNOM: Scattering scanning near-field optical microscopy
